# γ-Stearolactone ring-opening by zeolites for the production of branched saturated fatty acids†

**DOI:** 10.1039/d4cy00782d

**Published:** 2024-09-25

**Authors:** Jelle W. Bos, Job G. A. Vloedgraven, Sophie C. C. Wiedemann, Leo van Dongen, Roel C. J. Moonen, Bas Wels, Peter H. Berben, Bennie H. Reesink, Peter de Peinder, Eelco T. C. Vogt, Bert M. Weckhuysen

**Affiliations:** a Inorganic Chemistry and Catalysis Group, Institute for Sustainable and Circular Chemistry, Utrecht University Universiteitsweg 99 3584 CG Utrecht The Netherlands B.M.Weckhuysen@uu.nl; b Cargill Buurtje 1 2802 BE Gouda The Netherlands; c Symeres Vliesvenweg 1 6002 NM Weert The Netherlands; d BASF Nederland B.V. Strijkviertel 61 3454 PK Utrecht The Netherlands; e iQatalyst Strijkviertel 61 3454 PK Utrecht The Netherlands; f VibSpec Haaftenlaan 28 4006 XL Tiel The Netherlands

## Abstract

C_18_ branched saturated fatty acids (BSFA) are used as ingredients in cosmetics and lubricants and are produced *via* the hydrogenation of C_18_ branched unsaturated fatty acids (BUFA). Industrial-grade C_18_ BUFA contain the odorous by-product γ-stearolactone (GSL), which must be removed by acid-catalysed ring-opening of GSL into oleic acid. Zeolites such as Y and beta can facilitate the ring-opening, but due to the dimensions of GSL the activity is expected to be limited by diffusion into the micropores. Hence, zeolites Y and beta were modified *via* hydrothermal treatment and acid leaching and used in the GSL ring-opening reaction. While modification of zeolite beta led to a reduction in acidity of more than 50%, the material displayed much-enhanced activity compared to the parent material. In a batch reactor steamed beta zeolites were able to convert all GSL within 2 h, compared to 5 h for the parent zeolite. Infrared spectroscopy studies of adsorbed pyridine reveal that likely a beneficial change in Brønsted/Lewis acid site ratio is responsible for the increased activity. Lewis acid sites in zeolites are known to catalyse double bond isomerisation, which could greatly enhance GSL conversion by reducing the reverse formation of GSL from oleic acid. We believe that these insights can be used to further improve GSL ring-opening activity and inspire research on the ring-opening of other biomass derived lactones.

## Abstract 2

Improvement in γ-stearolactone ring-opening activity of beta zeolites by a favourable change in Brønsted to Lewis acid site ratio, induced by steaming and acid leaching.

## Graphical abstract

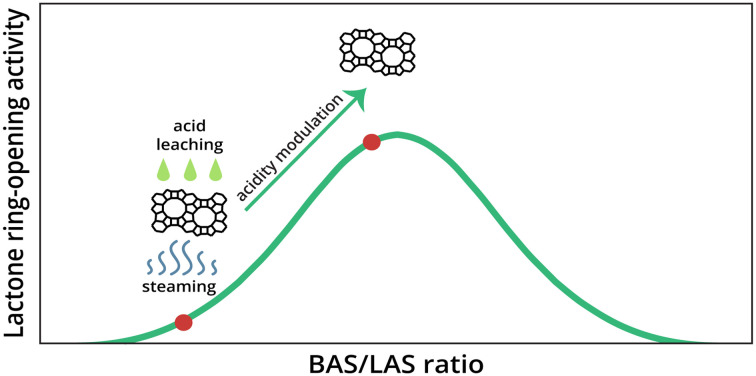

## Introduction

**Scheme 1:**
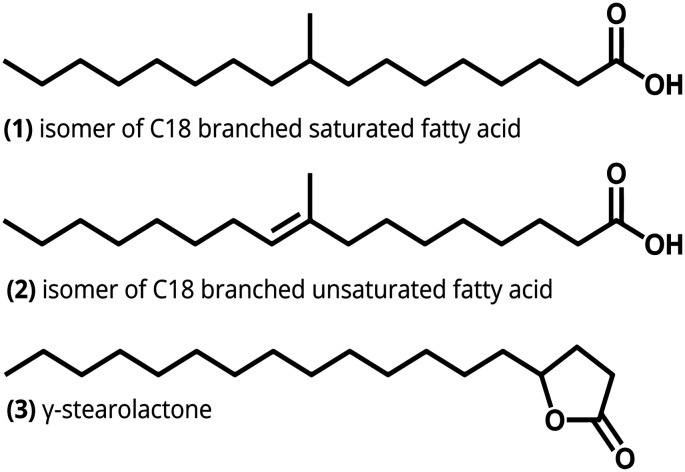
Structural formulas of isomers of the branched saturated and unsaturated fatty acids and the γ-stearolactone molecule, present in the C_18_ BUFA hydrogenation process.

C_18_ branched saturated fatty acids (BSFA) display unique physicochemical properties within the group of naturally derived fatty acids (FA). Being saturated FA, they are thermally and oxidatively stable and have a long shelf life.^1^ Furthermore, they display low-temperature liquidity and have a very good biodegradability. As such, C_18_ BSFA are frequently used in a wide variety of commercial applications, such as personal care products, lubricants and hydraulic fluids.^2,3^ The industrial production process starts with the hydrolysis of vegetable oils to obtain unsaturated fatty acids (UFA) and glycerol. After separation from the glycerol, the mixture of UFAs, consisting of mainly oleic acid (OAC), undergoes an acidic clay-catalyzed isomerization reaction to produce industrial-grade BUFA, the structure of one isomer is shown in Scheme 1. Due to its natural origin and double bond isomerization by the acidic catalyst, many positional isomers of the C_18_ BUFA are present in the isomerisation product. Apart from the isomerization to BUFA, intramolecular cyclization of the carboxyl group of OAC will occur when the carbocation is located at the 4-position.^4^ The product of this cyclization reaction is the odorous and oxidatively labile by-product γ-stearolactone (GSL), which must be removed from the reaction mixture. Permanent removal of GSL occurs when it is ring-opened by the acidic catalyst into OAC, and subsequently hydrogenated by a hydrogenating metal such as palladium. In this work zeolite catalyst candidates that could efficiently facilitate the ring-opening of GSL were evaluated.

A family of potential candidates as acid-based catalyst materials are zeolites. They are known for their intrinsic microporous nature and Brønsted acidity and therefore considered to be suitable candidates for the ring-opening of GSL in the presence of BUFA as size selectivity would impede the formation of fatty acid oligomers. Apart from the availability of a wide variety of different zeolite frameworks, their Si/Al ratio can also be tuned, and they are high surface area materials. More specifically, the 3-dimensional 12-membered ring (MR) zeolites Y (with framework structure FAU) and beta (with framework structure beta), which have large main pore dimensions relative to the GSL molecule, are deemed interesting candidates for this catalytic application. However, while zeolites Y and beta can likely facilitate the ring-opening, due to the large dimensions of GSL the activity might still be limited by the restricted diffusion within the zeolite micropores.

There are several studies in the open literature in which zeolite-catalyzed lactone ring-opening into unsaturated acids has been discussed, although these articles solely focus on the conversion of the much smaller biomass-derived γ-valerolactone (GVL).^5–9^ An example is the work by Al-Naji *et al.*, who have been using the 10-membered ring (MR) zeolite ZSM-5, stressing the importance of strong Brønsted acid sites (BAS) to facilitate the ring-opening reaction of GVL into pentenoic acids.^5^ On the contrary, Lange *et al.* report that ring-opening of GVL can be performed using zeolites or a variety of different strong and weak solid acids, and that the reaction seems not to be very demanding in terms of acid strength.^6^ Related to this, it has been described that Lewis acidic silver triflate is an efficient catalyst for the conversion of OAC into GSL, partly because of its double bond isomerization activity.^10^ Given these findings, it is likely that acidic properties other than just acid site strength play a part in GSL conversion. While research using zeolite-based materials in GVL ring-opening has been performed, to the best of our knowledge, the application of zeolites in GSL ring-opening is yet to be described. As such, this research set out to study the influence of variations in zeolite topology, porosity and acidity on GSL ring-opening activity.

More specifically in this work, first an initial screening of commercial 2-dimensional 10-MR ZSM-5, 1-dimensional 12-MR Mordenite and 3-dimensional 12-MR Y zeolites was performed to evaluate their suitability for GSL ring-opening during BUFA hydrogenation. After this screening it was decided to continue the exploration of the Y zeolite, and to compare it to 12-MR zeolite beta. Commercial zeolite Y and beta materials were then systematically modified by hydrothermal treatment and subsequent acid leaching to change the porosity and the acidity (*i.e.* the acid site distribution, density, and nature) of the zeolite materials. The modified zeolites were used in the catalytic hydrogenation of BUFA and their performance in GSL ring-opening has been compared with parent zeolite materials. Both sets of materials have been characterized by Ar-physisorption, Fourier Transform-Infrared (FTIR) spectroscopy of adsorbed pyridine and ^27^Al Magic Angle Spinning (MAS) Nuclear Magnetic Resonance (NMR) spectroscopy among others, thereby aiming to develop structure-composition-performance relationships for the GSL ring-opening reaction.

## Results and discussion

### Catalyst screening results

As GSL ring-opening by zeolites has never been described in literature, or systematically studied, first a quick screening of the two-dimensional 10-MR ZSM-5 (H-CBV2314), 1-dimensional 12-MR mordenite (H-MOR) and three-dimensional 12-MR Y (CBV-400) zeolites was performed to test their suitability for GSL ring-opening. The porous properties and acidity of these materials are summarized in Table S1,† the NH_3_-TPD profiles of the materials can be seen in Fig. S1a.† From the table it is clear that all these are high surface area materials, ranging from 351 (H-CBV2314), 433 (H-MOR) to 726 m^2^ g^−1^ (CBV-400). *T*-Plot analysis revealed that these are mostly microporous materials and that their mesoporosity is limited. Furthermore, NH_3_-TPD measurements showed that they are highly acidic in terms of number of acid sites, and moreover H-CBV2314 and H-MOR display large amounts of HT desorption, an indication of high acid strength. The materials were then tested on their GSL ring-opening activity in a BUFA hydrogenation experiment, the results of this can be seen in Fig. S1b.† GSL conversions of 73% (H-MOR), 80% (CBV-400) and 84% (H-CBV2314) were achieved with these zeolites in 5 h of reaction time. For H-CBV2314, a plateau of GSL conversion seems to have been reached over time, indicating possible deactivation of the zeolite. Compared to other studied catalysts, these materials were however not sufficiently active in GSL ring-opening, and are therefore deemed not suited for industrial application in their current form. It is likely that the reason for the low activity of these materials is that diffusion limitations of GSL occur, due to the limited mesoporosity of the zeolites. Of all the tested materials, zeolite Y (FAU topology) was chosen for follow-up experiments, as zeolites Y with increased mesoporosity are commercially available (USY). As a comparison to zeolite Y, the 3-dimensional 12-MR beta zeolite (beta topology) was also explored.

### Catalyst characterization

**Fig. 1:**
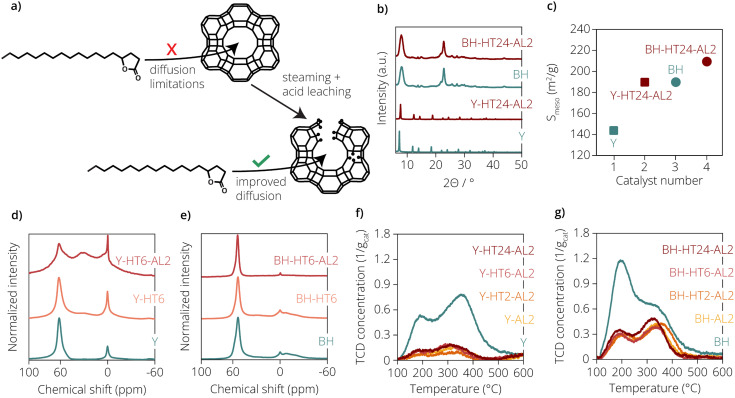
(a) Experimental approach to improve γ-stearolactone (GSL) conversion of microporous zeolites by removing their intrinsic diffusion limitations. (b) X-ray diffraction (XRD) patterns of the parent and most harshly treated zeolite Y and beta materials. In this fig. HT signifies hydrothermal treatment and the following number the duration of this treatment in hours. Furthermore, AL2 signifies a 2 M nitric acid washing treatment. (c) Mesopore surface area (*S*_meso_) values of the parent and most harshly treated zeolite Y and beta materials, determined by Ar-physisorption. (d) Normalized ^27^Al Magic Angle Spinning (MAS) Nuclear Magnetic Resonance (NMR) spectra of parent, 6 h steamed and steamed plus acid leached zeolite Y materials. (e) Normalized ^27^Al MAS NMR spectra of parent, 6 h steamed and steamed plus acid leached zeolite beta materials. (f) Temperature-based Temperature Programmed Desorption (TPD) profiles of all zeolite Y materials. (g) Temperature-based TPD profiles of all zeolite beta materials.

Two series of catalyst materials, based on zeolites Y (Alfa Aesar) and beta, were prepared *via* hydrothermal treatment and/or acid leaching, the experimental approach can be seen in Fig. 1a. In the naming of the catalyst, the number after HT stands for the number of hours of hydrothermal treatment the material underwent, whereas the number 2 after AL stands for the 2 M nitric acid treatment. Table 1 summarizes the names and physicochemical properties of the different zeolite materials, obtained after different preparation routes. The adsorption and desorption isotherms of the materials, obtained from N_2_-physisorption, can be seen in Fig. S2.†

**Table 1:** Sample names and physicochemical properties of the zeolite Y and beta materials under study

Sample	*S*_BET_ a (m^2^ g^−1^)	*S*_meso_ b (m^2^ g^−1^)	*V*_total_ c (cm^3^ g^−1^)	*V*_micro_ d (cm^3^ g^−1^)	Si/Al (bulk)^e^	Al content ratio^f^	Relative crystallinity^g^ (%)	*a*_0_ h (Å)
Y	775	143	0.48	0.24	17.7	1.00	100	24.32
Y-AL2	1168	284	0.76	0.34	119.1	0.15	88.5	24.22
Y-HT2-AL2	802	218	0.52	0.22	111.7	0.11	97.1	24.23
Y-HT6-AL2	759	218	0.57	0.20	104.7	0.19	100.1	24.24
Y-HT24-AL2	657	190	0.53	0.18	77.1	0.21	85.3	24.26
BH	510	190	0.82	0.12	14.4	1.00	100.0	—
BH-AL2	524	210	0.81	0.12	68.2	0.21	89.8	—
BH-HT2-AL2	497	198	0.77	0.12	65.8	0.22	101.3	—
BH-HT6-AL2	520	209	0.75	0.12	61.4	0.23	110.2	—
BH-HT24-AL2	521	210	0.74	0.12	55.0	0.26	104.5	—

a BET surface area determined from physisorption measurements.

b Mesopore surface area (*t*-plot) determined from physisorption measurements.

c Total pore volume determined from physisorption measurements.

d Micropore volume determined from physisorption measurements.

e From the Inductively Coupled Plasma-Optical Emission Spectroscopy (ICP-OES) measurements.

f Aluminium content ratio compared to parent zeolite, based on ICP-OES measurements.

g *Via* X-ray Diffraction (XRD) measurements.

h *Via* Rietveld refinement of the X-ray diffractograms.

Ar physisorption revealed that the parent Y zeolite already is significantly more mesoporous than the previously screened CBV-400 zeolite and displays a mesopore surface area (*S*_meso_) of 143 m^2^ g^−1^. Treatments of the Y zeolite applied during zeolite modification led to an increase in the mesoporosity of the materials. The mesopore surface area (*S*_meso_) reached a maximum value of ∼284 m^2^ g^−1^ for the Y-AL2 sample compared to a value of ∼143 m^2^ g^−1^ for the parent zeolite Y material. Also, the highest total surface area (*S*_BET_) is observed for the Y-AL2 zeolite (1168 m^2^ g^−1^). We believe these observations can be explained by the fact that this material was only acid-leached during this work. Any remaining extraframework aluminium (EFAL) species on the parent Y zeolite, would be removed by this procedure, thus increasing the porosity of the material. Also, for the steamed and acid leached zeolite Y materials increased mesopore surface areas were observed, although the effect was less pronounced than for the Y-AL2 sample. The parent beta zeolite also displays significant mesoporosity, with an *S*_meso_ of 190 m^2^ g^−1^. The differences in mesoporosity between the treated zeolite beta materials and the parent beta material were however much smaller than for the Y zeolites and can be considered within the margin of error of the physisorption measurements. It is believed that the limited increase in mesoporosity of the zeolite beta materials can in part be explained by the low aluminium content of the zeolite beta.

With a bulk Si/Al ratio of 14.4, steaming and/or acid leaching would only be able to remove a small fraction of the zeolite material *via* dealumination and as such the change in mesoporosity would be low.

Inductively Coupled Plasma-Optical Emission Spectroscopy (ICP-OES) analyses revealed that treatment of the zeolite Y and beta materials resulted in more strongly dealuminated zeolites. Both the Y-HT24-AL2 and BH-HT24-AL2 samples had the lowest Si/Al ratios of all the treated materials from their respective zeolite type. It is possible that this is due to the longer steaming time, which could lead to the formation of more highly polymerized extra-framework aluminium (EFAL) species. These EFAL species are more difficult to remove by acid leaching,^11^ and might thus still be present after leaching, explaining the higher Si/Al ratio determined *via* ICP-OES.

X-ray diffraction (XRD) results showed that the treatments in general led to a reduced crystallinity of the zeolite Y materials (with the Y-HT6-AL2 sample within margin of error). For the acid leaching of the zeolite beta materials a crystallinity decrease was observed, while steaming plus acid leaching led to an increased crystallinity of the zeolite material. In general, however, it was concluded that the treatments applied did not lead to significant damage to the zeolite framework structures. Rietveld analysis of the X-ray diffractograms of the Y zeolites was performed in order to determine the unit cell size *a*_0_ and verify zeolite framework dealumination. The observed, calculated and difference profiles of the refinements can be found in Fig. S4.† The residuals of the refinements can be found in Table S2.† These analyses showed that the residuals of the refinements were below 10%, so the fits between the observed and calculated profiles were deemed of high enough quality. It also revealed that Y-AL2 suffered the largest amount of framework dealumination, as evidenced by the largest reduction in unit cell size compared to the parent Y zeolite. It was also shown that the extent of framework dealumination of the steamed and acid-leached zeolites decreased with steaming time. This can likely be attributed to the progressive deposition of EFAL on the framework, which would protect it from acid leaching and prevent further framework dealumination. The XRD patterns of the parent and most harshly treated zeolite Y and beta materials are shown in Fig. 1b. For both the treated zeolite Y and beta materials, their respective characteristic zeolite framework reflections could be still observed. Apart from that, no amorphous material could be detected in the XRD patterns as the characteristic broad scattering intensity between 2*θ* values of 20 and 30°, due to amorphous silica(/alumina), was absent. Similar observations were made for the other treated zeolite materials, the XRD patterns of these samples can be seen in Fig. S3.† The changes in the local coordination of the aluminium atoms in the zeolite materials due to the treatments applied were monitored using ^27^Al Magic-Angle Spinning (MAS) Nuclear Magnetic Resonance (NMR) spectroscopy. The normalized NMR spectra of the three zeolite Y and three beta materials are shown in Fig. 1d and e respectively. Of the different steamed zeolite materials, only the 6 h steamed zeolites were analysed, it was expected that similar trends would be observed for the 2 h and 24 h variants. In the NMR spectra of the zeolite Y materials three distinct peaks could be distinguished, centered at ∼60, 30 and 0 ppm. The resonances at ∼60 and ∼0 ppm are attributed to aluminium atoms in a tetrahedral and octahedral coordination environment, respectively. The resonance at ∼30 ppm is attributed to pentahedral aluminium species.^11–13^ As expected, the NMR spectrum of the unmodified zeolite Y material showed peaks at ∼60 and ∼0 ppm. It was seen that upon 6 h of hydrothermal treatment, the spectrum of the Y-HT6 zeolite material displayed a slight peak at ∼30 ppm, indicating the presence of pentahedral aluminium species. These species are often deemed to be Lewis acidic EFAL species,^14^ and are generated by Al–O bond hydrolysis and the resulting expulsion of aluminium atoms from the zeolite framework during hydrothermal treatment. Acid leaching of the Y-HT6 sample did not seem to have the desired effect, as is seen in the spectrum of the Y-HT6-AL2 sample. While it was thought that acid leaching would lead to dissolution of the EFAL species and thus disappearance of the 30 ppm NMR peak, the height of the 30 ppm NMR peak relative to the other NMR peaks had instead increased strongly. Apart from that, the 60 ppm NMR peak seemed to have widened significantly, which could indicate the formation of distorted tetrahedral aluminium species.^15^ Such a statement would ideally have to be verified using 2D NMR spectroscopy, but this is beyond the scope of this research. It is however plausible that the acid leaching step was too severe for the zeolite Y material, attacking framework aluminium species, thus leading to distortions in the aluminium coordination. Other changes induced by the hydrothermal treatment and/or acid leaching procedures, such as the generation of defect sites on the zeolite frameworks, could in future work also be verified using ^29^Si MAS NMR.

In the NMR spectra of the zeolite beta materials also three NMR peaks could be identified, which are centered at ∼53, 30 and 0 ppm. Identical to the zeolite Y materials, these indicate the presence of tetrahedral, pentahedral and octahedral aluminium atoms, respectively.^16^ The NMR spectrum of the unmodified beta material displayed a large peak in the tetrahedral region and a small peak in the octahedral region. Apart from that, an additional broad feature was observed in the octahedral region. This broad feature signifies the presence of highly distorted octahedral aluminium atoms.^17,18^ Lastly, the NMR spectrum of the BH sample displayed significant intensity at ∼30 ppm, suggesting the presence of pentahedral aluminium atoms. Such an observation has been made before for unmodified zeolite beta material, and it is expected that this signal stems from EFAL species remaining after the zeolite synthesis.^16^ While difficult to observe on this scale, upon hydrothermal treatment of the BH sample a slight peak appeared at ∼30 ppm, confirming the formation of EFAL. After acid leaching this NMR peak had completely disappeared and therefore the procedure was successful in removal of the pentahedral EFAL species. Apart from that, the broad feature in the octahedral region had disappeared and the peak at ∼60 ppm had slightly narrowed. It was concluded that the acid-leaching step had the desired effect and that the treatments did not lead to distortions in the aluminium coordination. High Resolution-Transmission Electron Microscopy (HR-TEM) images of the Y-HT6-AL2 and BH-HT6-AL2 samples can be seen in Fig. S5a and S5b,† respectively. For the Y-HT6-AL2 sample, large mesopore channels were clearly visible on the zeolite crystal, as shown by the lighter parts in the zeolite. These pores were likely already present in the parent zeolite Y material (with a Si/Al ratio of 15) as a result of the steaming and acid leaching required to produce ultrastable Y (USY) from low Si/Al ratio zeolite Y materials. Although EFAL species were detected in NMR, mesopores of smaller dimensions formed by the applied hydrothermal treatment were not visible on the zeolite. Visible in the image were the lattice fringes of the zeolite, which confirmed the presence of crystalline domains in the inspected sample. The HR-TEM image of the BH-HT6-AL2 sample revealed small crystals around 10–20 nm in size, seemingly clustered together. No mesopores could be distinguished in the crystals of the BH-HT6-AL2 sample, so its mesoporosity, determined by Ar-physisorption, is attributed to inter-crystalline voids.

To study the effect of the hydrothermal and acid treatments on the acidity of the zeolites, NH_3_-Temperature Programmed Desorption (NH_3_-TPD) experiments were performed. The NH_3_-TPD profiles of the studied zeolite Y and beta materials are given in Fig. 1f and g, respectively. As it was suspected that above 600 °C dehydroxylation of the zeolite materials was taking place, the profiles are only shown up to 600 °C. The total acidity of the different zeolites, determined by integration of the TPD profiles, can be found in Table 2. The ratio between the total acidity of the parent zeolites and their modified variants, can also be seen in Table 2.

**Table 2:** Acidity of all studied zeolite Y and beta materials in mmol g_cat_^−1^, determined by NH_3_-Temperature Programmed Desorption (NH3-TPD). The acidity in the low (LT), intermediate (IT) and high temperature (HT) regimes are given separately from the total acidity

Sample	Acidity (mmol g^−1^)				
	LT (100–250 °C)	IT (250–400 °C)	LT (400–600 °C)	Total	Acidity ratio
Y	0.15	0.26	0.08	0.49	1.00
Y-AL2	0.02	0.05	0.01	0.07	0.15
Y-HT2-AL2	0.02	0.03	0.01	0.05	0.11
Y-HT6-AL2	0.03	0.05	0.01	0.09	0.19
Y-HT24-AL2	0.03	0.05	0.01	0.10	0.21
BH	0.30	0.24	0.06	0.60	1.00
BH-AL2	0.07	0.13	0.02	0.22	0.37
BH-HT2-AL2	0.08	0.13	0.02	0.24	0.39
BH-HT6-AL2	0.08	0.12	0.01	0.20	0.33
BH-HT24-AL2	0.10	0.14	0.01	0.25	0.42

In the TPD profile in Fig. 1f we could distinguish two peaks for the unmodified zeolite Y material, a low temperature (LT) peak at ∼195 °C and an intermediate temperature (IT) peak at ∼355 °C. For the modified zeolite Y materials, the LT peaks could also be found at ∼195 °C, but the IT peaks had shifted to temperatures between 300 and 310 °C. High temperature (HT) desorption was also observed, and this occurred above 400 °C. The profile of the unmodified zeolite Y material showed high areas for the LT and IT desorption peaks. Upon treatment of the zeolite with 2 M nitric acid or additionally with steam, the areas of the LT and IT peaks were strongly diminished. Similarly, the area of the HT desorption peak of the modified zeolite Y materials had strongly decreased compared to that of the unmodified zeolite Y material. While small differences were observed, the Y-AL2, Y-HT2-AL2, Y-HT6-AL2 and Y-HT24-AL2 samples all displayed comparable LT, IT and HT acidity. It was concluded that the acid-leaching step was severe and led to a large decrease in the total acidity of the zeolite material. Considering that previous research stated the need for strong Brønsted acidity for lactone ring-opening, these results were however interesting. As the modified zeolite Y material displayed strongly reduced acidity, it was expected that these zeolites would be poor GSL ring-opening catalysts. Comparing the acidity ratio of the Y zeolites with their aluminium content ratio determined using ICP-OES (given in Table 1), we see that these match quite well.

In the TPD profiles of the zeolite beta materials also two peaks could be distinguished. An LT peak was observed for the unmodified zeolite beta at ∼195 °C and an IT peak at ∼340 °C was observed. For the modified zeolite beta materials, the LT peaks were observed between 190 and 210 °C and the IT peaks were observed between 325 and 355 °C. The profile of the unmodified zeolite beta material displayed the largest LT peak area and furthermore a large IT area, with only small amounts of NH_3_ desorbing at higher temperatures. For the treated zeolite beta materials, a much different acid site distribution was observed. Most notably, the LT peak areas had strongly decreased, but also the IT peak area was reduced. As a result, now not the LT, but the IT desorption was most prominent in the TPD profiles of the zeolite materials. Similar to the modified zeolite Y materials, little HT desorption was observed for the modified zeolite beta materials. In general, it was noted that a much higher fraction of the original total acidity was retained in the zeolite beta materials after modifications as compared to the zeolite Y materials. In the case of the beta zeolites, the acidity ratios are significantly lower than the aluminium content ratios. It is believed that the presence of EFAL species on the parent beta zeolite is the cause for this deviation. As EFAL species cover the acid sites of the parent beta zeolite, the acidity of the material is underestimated, leading to an underestimation of the acidity ratio and lower value compared to the aluminium content ratio.

**Fig. 2:**
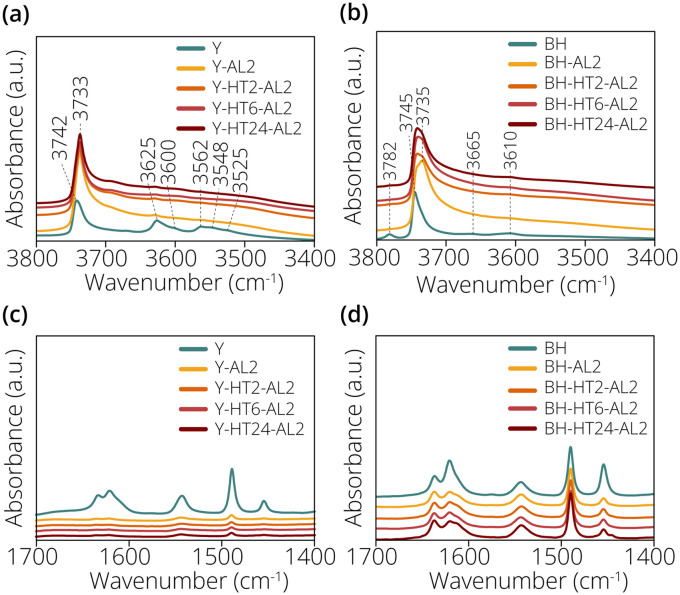
Fourier transform-infrared (FT-IR) spectra of the OH stretching region of (a) the zeolite Y materials prior to pyridine adsorption and (b) the zeolite beta materials prior to pyridine adsorption. FT-IR spectra post pyridine adsorption of (c) the zeolite Y materials and (d) the zeolite beta materials.

Additional characterization of the zeolite acidity was performed with Fourier Transform Infrared (FT-IR) spectroscopy of adsorbed pyridine. The OH stretching region of the FT-IR spectra of the zeolite Y materials prior to adsorption of pyridine is shown in Fig. 2a.

The IR bands observed at ∼3742 cm^−1^ and ∼3733 cm^−1^ for the modified Y zeolites were attributed to silanol groups.^19^ The IR bands at ∼3625 and ∼3562 cm^−1^ can be attributed to high frequency (HF) and low frequency (LF) bridging hydroxyls inside the supercage and sodalite cage respectively. The IR band at ∼3600 cm^−1^ can also be attributed to an HF bridging hydroxyl species, perturbed by EFAL species created by the steam dealumination.^11^ The IR band at ∼3548 cm^−1^ can be attributed to another bridging hydroxyl inside the sodalite cage.^20^ The IR band at ∼3525 cm^−1^ is attributed to LF bridging hydroxyl groups interacting with EFAL species.^21^ The OH stretching region of the FT-IR spectra of the zeolite beta materials prior to adsorption of pyridine is shown in Fig. 2b. The IR band observed at ∼3782 cm^−1^ for the unmodified zeolite beta material is attributed to aluminol groups.^22^ This band was still visible after introduction of pyridine, and a part of these acid sites can thus apparently not be reached by pyridine. The IR band at ∼3745 cm^−1^ is assigned to terminal silanol groups, whereas the IR band at ∼3735 cm^−1^ could be assigned to isolated internal silanol groups.^23,24^ The IR band at ∼3665 cm^−1^ is assigned to AlO–H groups and that at ∼3610 cm^−1^ is assigned to the bridging hydroxyl groups.^25,26^ The FT-IR spectra of zeolite Y and beta materials after adsorption of pyridine are shown in Fig. 2c and d, respectively. For the unmodified zeolite Y material distinct IR bands at ∼1543 and ∼1454 cm^−1^ were observed, which indicated the presence of BAS and LAS, respectively.^27^

Upon treatment of the zeolite materials the intensity of all the IR bands involving pyridine vibrations decreased strongly. This showed that the treatments had a detrimental effect on the overall acidity of the zeolite materials, removing large quantities of both BAS and LAS. In the FT-IR spectra of the zeolite beta materials shown in Fig. 2d, IR bands were observed at ∼1543 and ∼1454 cm^−1^, again indicating the presence of BAS and LAS. A marked decrease in the area of the 1454 cm^−1^ band was observed upon treatment of the zeolite beta materials, indicating a decrease in their Lewis acidity. Contrary to the zeolite Y zeolites, however, the areas of the IR bands indicating the presence of BAS and LAS remained significant after treatment.

**Fig. 3:**
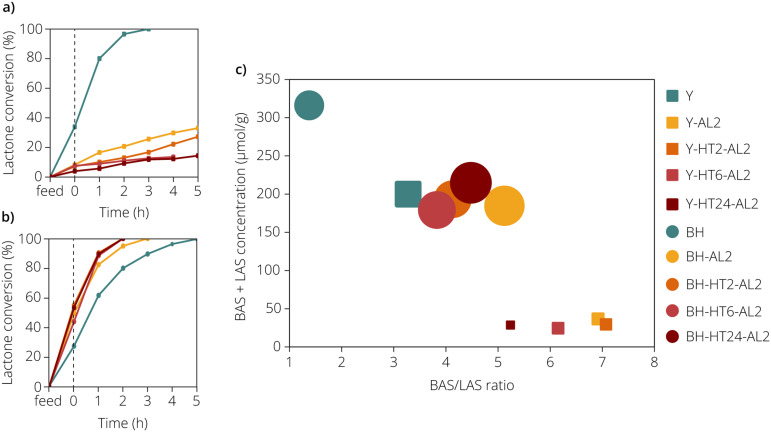
γ-Stearolactone (GSL) ring-opening activities of the different (a) zeolite Y and (b) zeolite beta materials under study, as summarized in Table 1. The elapsed time before the dashed line (*t* = 0 h) is the time needed to heat from room temperature to the final temperature of 230 °C. (c) Calculated total acidity (μmol g^−1^) and Brønsted acid site to Lewis acid site (BAS/LAS) ratios of the zeolite materials as probed by pyridine in combination with Fourier transform-infrared (FT-IR) spectroscopy. The sample symbols have been adapted so that the area of each symbol is proportional to the GSL conversion up to when the target temperature of 230 °C (*t* = 0 h) was reached.

The calculated total acidity and the Brønsted acid to Lewis acid site ratio (expressed as the BAS/LAS ratio) as probed by pyridine are given in Fig. 3c for all the studied zeolite materials. These calculations were based on the molar absorption coefficients of BAS and LAS at an analyte temperature of 150 °C, as determined by Zholobenko *et al.*^28^ For the zeolite Y materials, the molar absorption coefficients are 1.54 and 1.71 cm μmol^−1^ for BAS and LAS, respectively. For the zeolite beta materials, the molar absorption coefficients are 1.12 and 1.71 cm μmol^−1^ for BAS and LAS, respectively.

The unmodified zeolite Y material possesses a total acidity of 200 μmol g^−1^ and a BAS/LAS ratio of 3.3. For zeolite Y, a discrepancy in total acidity was observed where the total acidity as probed by pyridine was about 2.5 times lower than that probed by ammonia. It has already been reported that pyridine cannot access the sodalite cage of zeolite Y, while ammonia can.^29,30^ It is therefore likely that this discrepancy in acidity is at least partly caused by the inability of pyridine to probe the acid sites inside the sodalite cage of zeolite Y (that may also be inaccessible to the reactant molecules). As was expected from the IR spectra and the NH_3_-TPD measurements, the modified zeolite Y materials displayed strongly reduced total acidity, ranging between 24 and 36 μmol g^−1^. The BAS/LAS ratios of these zeolite materials varied between 5.2 and 7.1. With such low acidity and high BAS/LAS ratios, the modified zeolite Y materials contained virtually no Lewis acid sites. The unmodified zeolite beta material displayed the highest total acidity at 317 μmol g^−1^ and the lowest BAS/LAS ratio at 1.38. Notably, the LAS concentration of this sample was at least 3 times higher than the LAS concentration of any of the other zeolite material under study. Upon modification of the zeolite beta materials, the total acidity was reduced to values between 215 and 180 μmol g^−1^. This was a marked reduction in total acidity, but contrary to the zeolite Y materials still significant acidity was retained. The BAS/LAS ratios of the modified zeolite beta materials ranged between 3.82 and 5.12.

### Catalytic testing

All studied zeolite Y and beta materials were tested on their GSL ring-opening activity in BUFA hydrogenation experiments. The GSL ring- opening activity of the various zeolite Y materials are summarized in Fig. 3a. The concentrations of GSL (in wt%) determined in each sample, can be seen in Table S3.† The parent zeolite Y material vastly outperformed the treated zeolite Y materials, having converted all of the lactone in 3 h. The second most active catalyst, namely the Y-AL2 sample, only converted 33% of the γ-lactone in 5 h of hydrogenation. The activity was diminished even further for the steam-treated zeolites, with lactone conversion after 5 h slumping to 14% for the Y-HT24-AL2 sample. The GSL ring-opening activity of the various zeolite beta materials is shown in Fig. 3b. In contrast to the zeolite Y materials, the parent zeolite beta was the least active catalyst of all, requiring 5 h to fully convert the lactone. Upon treatment with concentrated acid the activity was increased significantly, as the BH-AL2 sample enabled full conversion in 3 h. An additional improvement was observed for all three steamed and acid-washed zeolites, with full conversion being reached in merely 2 h. Interestingly, for the treated zeolite beta material already around 50% of the lactone had been converted before reaching the final temperature of 230 °C (*t* = 0 h). This shows that the lactone ring-opening reaction can be performed at markedly lower temperatures than the selected temperature, which could simultaneously reduce the energy consumption for the production of C_18_ BSFA and improve catalyst stability. When the acidity, as measured with FT-IR spectroscopy with pyridine as probe molecule was related to the activity of the catalysts (Fig. 3c) we observed that the most active zeolites, the treated zeolite beta materials, have BAS/LAS ratios that ranged between 3.8 and 5.1.

ICP-MS measurements performed on the reaction product revealed significant leaching of aluminium from the Y zeolite. The total amount of aluminium present in the product signalled an 8% loss of total aluminium content from the Y zeolite. The modified Y zeolites were resistant to leaching induced by the medium, as the concentrations of aluminium detected in the product were similar to the feedstock concentration. For the BH zeolite, the total amount of aluminium present in the product represented a 21% loss in aluminium content. Similar to the Y zeolites, the treated beta zeolites were resistant to leaching, with aluminium concentrations in the product being very low. It is believed that the improved stability of the zeolites can be attributed to the strong acid leaching procedure, which is thought to remove less strongly bound aluminium atoms from the zeolite structure. As these are already removed prior to testing, the more weakly acidic fatty acid is not capable of removing significant amounts of aluminium. Furthermore, high-temperature gas chromatography (HT-GC) revealed only minor amounts of oligomeric fatty acids in the reaction products. All iodine values of the reaction products were very low, confirming complete hydrogenation of the unsaturated fatty acids. The determined saponification and acid values were also within the norm. All the values determined *via* inductively coupled plasma mass spectrometry, iodine value, saponification value, acid value and HT-GC analyses can be found in Table S4 in the ESI.†

Despite the increased mesoporosity, reduced activity was observed for the modified Y zeolites. This reduction can likely be explained by the strongly lowered acidity of these zeolites. Especially the amount of strong acid sites was reduced as a result of the treatments applied, which would explain the low activity if these sites are indeed as crucial in lactone ring-opening as literature describes.^5^ More curious is the observed low activity of the parent zeolite beta material, as of all zeolite beta materials it boasted the highest concentration of both BAS and LAS and furthermore showed the highest HT desorption peak in the NH_3_-TPD measurements. Moreover, physisorption showed that this zeolite material furthermore had a high mesopore surface area. Similarly, the much-enhanced activity of the treated zeolite beta materials is unexpected, as these zeolite materials showed almost no HT desorption peak in the NH_3_-TPD measurements, even though strong acid sites have previously been reported to be essential for high lactone ring-opening activity.^5^ Furthermore, significant increases in the mesoporosity of the zeolite beta materials as a result of the treatments were absent. It therefore seems that strong acidity is not a trustworthy indicator for high GSL ring-opening activity. The higher activity of the treated zeolite beta materials might instead be explained by a change in the BAS/LAS ratio. The role of Lewis acid sites in GSL conversion is unknown, but these sites could play an important part. The product of the GSL ring-opening process is an OAC molecule with the double bond at the 4-position with respect to the carboxyl group. Naturally, if the double bond remains at this position, upon formation of a carbocation this OAC molecule is likely once more transformed into GSL. If, however, the double bond were to migrate away from the 4-position, this reduces the likelihood of GSL reformation and would increase GSL conversion. LAS in zeolite materials have previously shown to be capable of double isomerization, such as in gaseous n-butene.^31,32^ Moreover, double bond migration over ferrierite (FER) has been observed for OAC, although this behaviour has not specifically been attributed to the presence of LAS.^33^ If LAS in the studied zeolite materials cause significant double bond isomerization during BUFA hydrogenation, then modulation of the BAS/LAS ratio is one way to further improve GSL ring-opening activity. Furthermore, this would also implicate that purely Brønsted acidic zeolite materials are not ideal GSL ring-opening catalyst materials.

## Experimental section

### Catalyst preparation, chemicals and materials

Zeolite Y (in its H^+^ form, with a Si/Al ratio of 15, and a surface area of 780 m^2^ g^−1^), zeolite beta (in its NH_4_^+^ form, with a Si/Al ratio of 12.5, and surface area of 680 m^2^ g^−1^) and zeolite Mordenite (in its NH_4_^+^ form, with a Si/Al ratio of 9.5, and surface area of 598 m^2^ g^−1^) were supplied by Alfa Aesar. Zeolite Y (CBV-400, in its H^+^ form, with a Si/Al ratio of 2.55, and surface area of 730 m^2^ g^−1^) and zeolite ZSM-5 (CBV-2314, in its NH_4_^+^ form, with a Si/Al ratio of 11.5, and surface area of 425 m^2^ g^−1^) were supplied by Zeolyst. Pd/C (5 wt%) was supplied by BASF. Nitric acid (65%) was bought from Sigma Aldrich. Industrial samples of C_18_ branched unsaturated fatty acids (BUFA) and C_18_ branched saturated fatty acids (BSFA) were supplied by Cargill (operating by the name Croda at the time of the start of the research project).

Prior to modifications, zeolites beta and Mordenite were calcined in air in a muffle furnace to obtain the H^+^ form of the zeolite material (further denoted as BH and H-MOR respectively). The temperature was first increased with 1 °C min^−1^ until a temperature of 550 °C was reached. The zeolite material was then calcined at 550 °C for 6 h before being cooled down to room temperature. Zeolite CBV-2314 was first heated in a static oven to 100 °C and kept at this temperature for 1 h. It was then heated with 100 °C h^−1^ to the calcination temperature of 500 °C and calcined at 500 °C for 5 h to obtained the proton form of the zeolite (further denoted as H-CBV2314), after which it was cooled to room temperature. Zeolite Y (Alfa Aesar) and Zeolite Y (Zeolyst) were used in their supplied form without calcination (further denoted as Y and CBV-400, respectively).

The Y (Alfa Aesar) and beta zeolite materials under study were hydrothermally treated by placing 1 g of the H-form zeolite in a quartz calcination boat inside a Carbolite Gero tubular oven. The oven was then heated to 500 °C at a rate of 10 °C min^−1^ and at the same time a flask with distilled water was heated to ∼90 °C to create an 80% humidity atmosphere. The steam was then led through the reactor by applying a flow of 45 mL min^−1^ of N_2_ (Linde, >99.9%). Thereby samples named Y-HT2, Y-HT6, Y-HT24, BH-HT2, BH-HT6 and BH-HT24 were obtained, the number in the name indicating the time spent steaming at 500 °C. Acid leaching was performed by stirring the zeolite at 500 rpm in a 2 M nitric acid solution at 60 °C for 2 h. The leached zeolites were then washed with demineralized water and dried in a 60 °C oven overnight. The zeolite materials obtained are denoted as Y-HT2-AL2, Y-HT6-AL2, Y-HT24-AL2, BH-HT2-AL2, BH-HT6-AL2 and BH-HT24-AL2, with AL indicating the acid leaching step and 2 indicating the 2 M nitric acid used for this treatment.

### Catalyst characterization

Specific surface areas of the zeolite samples were analysed using physisorption of argon at 87 K using a 3P Sync400 physisorption machine. Prior to the measurements, samples were degassed under vacuum at 400 °C for 16 h. The total surface area (*S*_BET_) was determined according to the Brunauer–Emmett–Teller (BET) theory.^33^ The mesopore surface area (*S*_meso_) was determined *via* the empirical *t*-plot method using the Harkins Jura method.^34^ X-ray Diffraction (XRD) measurements were performed on a Bruker 2nd Gen D2 Phaser instrument using a Co source with a wavelength of 1.7902 Å. XRD patterns were recorded at 295 K between 2*θ* values of 5° and 50° with a step size of 0.04° and 0.5 s scan speed. The relative crystallinity of the treated zeolite Y materials was determined by summing the integrated intensities of the characteristic FAU reflections at 7.2, 11.9, 14.0, 18.4, 22.0, 24.0, 27.9 and 31.9° and dividing this by the sum of integrated intensities of the parent zeolite Y material. The crystallinity of the treated zeolite beta materials was determined in a similar way by integrating the characteristic BEA reflections at 8.0 and 22.8°. Dealumination of the zeolite frameworks was verified by calculation of the unit cell size *a*_0_*via* Rietveld refinement of the X-ray diffractograms. Calculations were performed in the GSAS II software kit, taking a chemical formula of Si_176_Al_22_O_406_ and space group F d 3̄*m* for ultrastable Y (USY), and a *Z*-value (charge) of 0. The number of reflections included into the refinements varied between 50 and 55. As method for background treatment, the Chebyschev-1 method was employed.

Inductively Coupled Plasma-Optical Emission Spectroscopy (ICP-OES) measurements to determine the Si/Al ratios were performed by Mikrolab Kolbe according to an in-house procedure. Ammonia Temperature-Programmed Desorption (NH_3_-TPD) measurements were performed on a Micromeritics Autochem II Chemisorption Analyzer equipped with a Thermal Conductivity Detector (TCD) detector. Prior to adsorption of ammonia, zeolites samples of 80–120 mg were heated to 600 °C with a rate of 5 °C min^−1^ and dried for 60 min at 600 °C to remove any adsorbed species from the zeolite material. Then an excess of ammonia was pulsed, to fully saturate the sample. Lastly, the zeolite was heated to 700 °C at a heating rate of 5 °C min^−1^ and ammonia was left to desorb at 700 °C for a further 30 min. The acidity of the different zeolite materials under study was further probed by Fourier Transform-Infrared (FT-IR) spectroscopy of adsorbed pyridine. FT-IR spectra were recorded on a Thermo Fisher iS5 FTIR spectrometer with a CaF_2_ window. Spectra were recorded with a resolution of 4 cm^−1^ and a total of 32 scans. Zeolite pellets were prepared by pressing 10–20 mg of zeolite powder onto a 1.3 cm^2^ pressing die, these were then brought into a transmission cell. Prior to the adsorption of pyridine, the pellets were dried for 2 h at 550 °C *in vacuo* to remove all adsorbed species from the zeolites. After cooling down to room temperature, a pre-adsorption spectrum of the dry pellet was recorded. Pyridine was then dosed at room temperature and the system was left to equilibrate for 30 min. Afterwards, the cell was heated to 150 °C at a ramp of 2.5 °C min^−1^, excess pyridine was desorbed for 30 min and lastly a spectrum was recorded. This spectrum, alongside the pre-adsorption spectrum, was used to calculate the total acidity of the zeolites. To obtain spectra that could be fairly compared with each other, all spectra were normalized with respect to the overtones of the zeolite lattice vibrations (between 2113 and 1872 cm^−1^). The zeolite materials were also analysed with ^27^Al Magic Angle Spinning (MAS) solid-state Nuclear Magnetic Resonance (NMR) spectroscopy on a Bruker Avance III spectrometer operating a magnetic field strength of 11.7 T and 500 MHz. The spectrometer was equipped with a 3.2 mm triple-resonance probe head with thin wall ZrO_2_ rotors, spinning at a MAS rate of 15 kHz. The ^27^Al chemical shift values were determined with the help of a saturated Al(NO_3_)_3_ solution. High-Resolution Transmission Electron Microscopy (HR-TEM) images were taken on a Tecnai F20 microscope, operating at an acceleration voltage of 200 kV. Prior to HR-TEM measurements, the samples were finely ground using a pestle and mortar and then sonicated in ethanol for 30 s. A droplet of the suspended zeolite material was then deposited on a TEM grid and the grid was air-dried.

### Catalytic testing and product analysis

Prior to catalytic testing, the zeolite powders were pressed into pellets, crushed, and lastly sieved in a size fraction of 212–425 μm. A 200 g fatty acid mixture consisting of 40 wt% industrial grade C_18_ branched unsaturated fatty acids (BUFA) and 60 wt% industrial grade C_18_ branched saturated fatty acid (BSFA) was prepared and added to a stirred autoclave reactor. The γ-stearolactone (GSL) concentration in the mixtures then ranged between 2.29 and 2.42 wt%, depending on the BUFA feedstock used. Afterwards, 0.5 g of the sieved zeolite material and 0.25 g of a Pd/C catalyst were added to the reactor. The system was then closed and flushed three times with N_2_ and H_2_ to remove any oxygen molecules. The reactor was stirred at 600 rpm and a H_2_-pressure of 15 bar was applied. The temperature was increased by 2 °C min^−1^ until a temperature of 190 °C was reached. Then, the ramp was increased to 4 °C min^−1^ and the pressure was increased to 20 bar. Once a temperature of 230 °C was reached, the pressure was finally set to 25 bar. Samples were taken from the reactor at regular intervals to be able to follow the ring-opening and hydrogenation reactions as a function of reaction time. The reactants and reaction products were extensively analyzed by determining the lactone content, iodine value, acid value, saponification value, fatty acid composition, monomer content and aluminum content. The detailed procedures of these analytical measurements can be found in the (ESI†).

## Conclusions

Zeolite materials, more specifically zeolite Y and beta, have been modified *via* both hydrothermal treatment and acid leaching and subsequently applied in the ring-opening reaction of γ-stearolactone (GSL). Physisorption results revealed a significant increase in the mesoporosity of the treated zeolite Y materials, while no noticeable changes in porosity were observed for the zeolite beta materials. It was found that modification of zeolite Y materials led to a strong decrease in their overall acidity and their activity was greatly diminished compared to the parent zeolite Y material. For the zeolite beta materials, the applied treatment steps also led to a reduced acidity, but contrary to the zeolite Y materials also to a strongly increased activity. Infrared spectroscopy studies support the conclusion that a change in the Brønsted acid site (BAS)/Lewis acid site (LAS) ratio contributed to this improved catalytic activity. LAS in zeolites are known to cause double bond isomerisation, a property that could greatly enhance the GSL conversion by reducing the reformation of GSL from oleic (OAC). To improve the GSL ring-opening activity more, further work should focus on the creation of zeolite materials with even more pronounced mesoporosity, for example *via* a desilication process. Care should be taken so that such modifications are not detrimental to the accessibility of the acid sites in the materials. It is believed that the findings of this work can inspire research on the combined ring-opening and hydrogenation of other biomass-derived lactones.

## Data availability

The data supporting this article have been included as part of the ESI.†

## Author contributions

J. W. B. co-conceptualized the project, performed the experiments, analysed the data, supervised the experiments by other members of the team, and wrote the initial version of the manuscript. J. G. A. V. prepared and analysed the catalyst materials. S. C. C. W. co-conceptualized the project, facilitated experiments at Cargill and provided discussions and supervised throughout the project. L. D. performed the catalytic tests at Cargill. R. M. and B. W. provided resources, facilitated experiments at Cargill and provided discussions. P. H. B. provided resources and discussions to the project. B. R. provided discussions to the project. P. P. provided resources and discussions to the project. E. T. C. V. provided discussions and supervised throughout the project and writing of the manuscript. B. M. W. co-conceptualized the project, provided resources, discussions, and validation, supervised throughout the project, and was involved in the writing of the manuscript. All authors have commented and provided input on the final version of the manuscript.

## Conflicts of interest

The authors declare no conflict of interest.

## Supplementary Material

CY-014-D4CY00782D-s001
