# Supplementary material for: γ-Stearolactone ring-opening by zeolites for the production of branched saturated fatty acids
Source: Catal Sci Technol. 2024 Sep 25;14(24):7037–47. doi: 10.1039/d4cy00782d (PMC11589803; doi:10.1039/d4cy00782d)
Supplement: CY-014-D4CY00782D-s001 [file CY-014-D4CY00782D-s001.pdf]

Supplementary Information (SI) for Catalysis Science & Technology.
This journal is © The Royal Society of Chemistry 2024

γ-Stearolactone Ring-Opening by Zeolites for the Production of Branched Saturated Fatty Acids

Jelle W. Bos,[a] Job G.A. Vloedgraven,[a] Sophie C.C. Wiedemann,[b] Leo van Dongen,[b] Roel C.J. Moonen,[b] Bas Wels,[c] Peter H. Berben,[d] Bennie H. Reesink,[d,e] Peter de Peinder,[a,f] Eelco T.C. Vogt,[a] and Bert M. Weckhuysen[*a]

[a] Inorganic Chemistry and Catalysis group, Institute for Sustainable and Circular Chemistry, Utrecht University, Universiteitsweg 99, 3584 CG Utrecht, The Netherlands; Email: B.M.Weckhuysen@uu.nl

[b] Cargill, Buurtje 1, 2802 BE Gouda, The Netherlands

[c] Symeres, Vliesvenweg 1, 6002 NM Weert, the Netherlands

[d] BASF Nederland B.V., Strijkviertel 61, 3454 PK Utrecht, The Netherlands

[e] iQatalyst, Strijkviertel 61, 3454 PK Utrecht, The Netherlands

[f] VibSpec, Haaftenlaan 28, 4006 XL Tiel, The Netherlands

1. CATALYST SCREENING RESULTS

Table S1. Porous properties and acidity of the screened ZSM-5 (H-CBV2314), Mordenite (H-MOR) and Y (CBV-400) zeolites. The acidity in the low (LT), intermediate (IT) and high temperature (HT) regimes are given separately from the total acidity.

	Porous properties				Acidity ($mmol/g_{cat}$)[e]			
Sample name	S_{BET} (m^2/g)[a]	S_{meso} (m^2/g)[b]	V_{total} (cm^3/g)[c]	V_{micro} (cm^3/g)[d]	LT (100-250 °C)	IT (250-400 °C)	HT (400-600 °C)	Total
H-CBV2314	351	37	0.18	0.11	0.47	0.40	0.29	1.16
H-MOR	433	50	0.23	0.14	0.38	0.21	0.49	1.08
CBV400	726	90	0.36	0.23	0.41	0.30	0.06	0.77

[a] BET surface area determined from physisorption measurements.
[b] Mesopore surface area (t-plot) determined from physisorption measurements.
[c] Total pore volume determined from physisorption measurements.
[d] Micropore volume determined from physisorption measurements.
[e] Acidity of the material in $mmol/g_{cat}$, determined by NH_3-Temperature Programmed Desorption (NH_3-TPD).

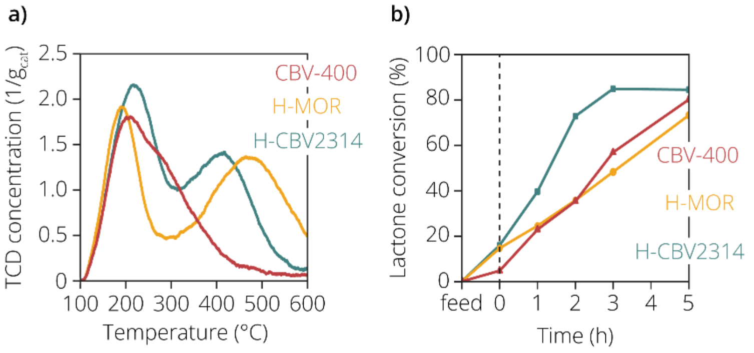

Figure S1. (a) Temperature-based temperature programmed desorption (TPD) profiles of the screened ZSM-5 (H-CBV2314), Mordenite (H-MOR) and Y (CBV-400) zeolites (b) γ-stearolactone (GSL) ring-opening activities of the screened ZSM-5 (H-CBV2314), Mordenite (H-MOR) and Y (CBV-400) zeolites. The elapsed time before the dashed line (t = 0 h) is the time needed to heat from room temperature to the final temperature of 230 °C.

2. ADDITIONAL CATALYST CHARACTERIZATION RESULTS

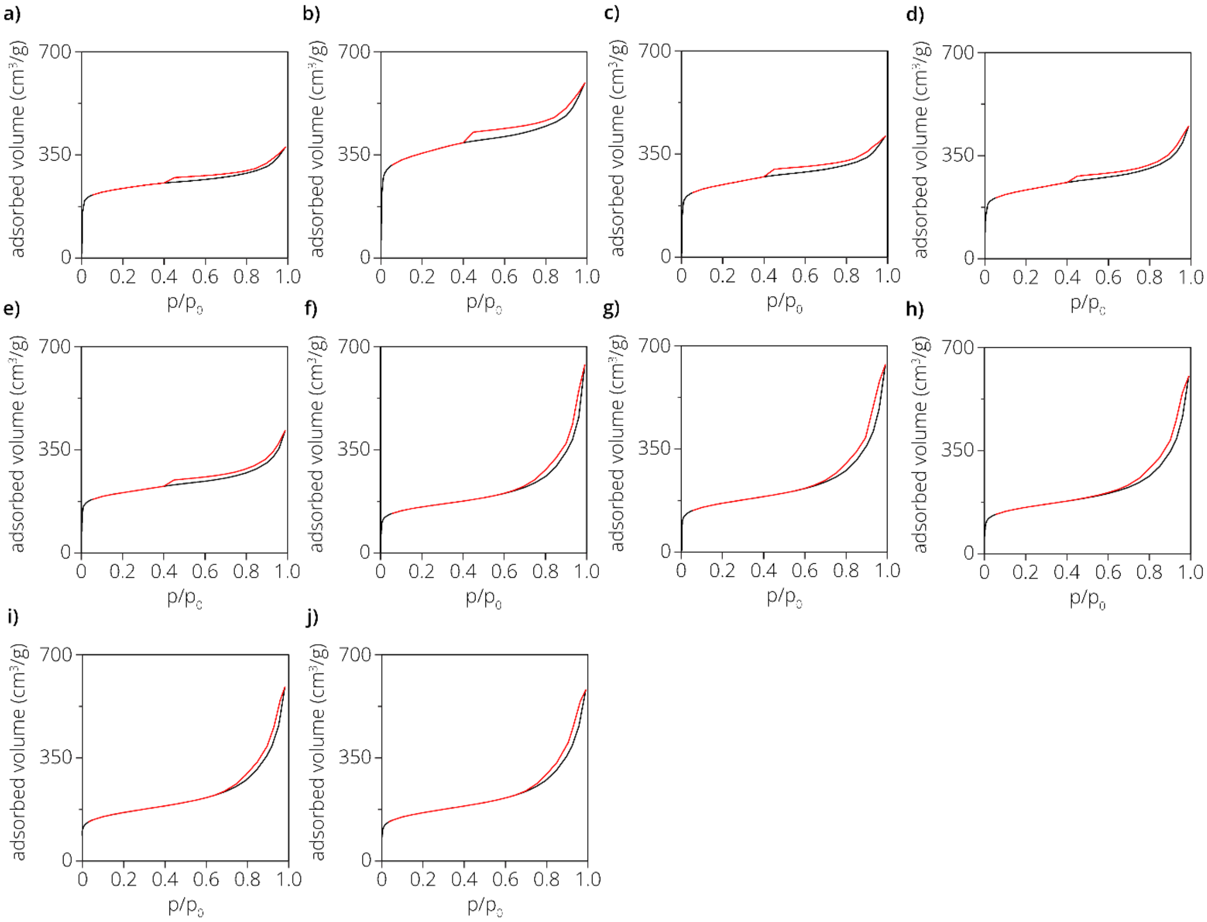

Figure S2. Nitrogen physisorption adsorption and desorption isotherms of the Y, Y-AL2, Y-HT2-AL2, Y-HT6-AL2, and Y-HT24-AL2. (a-e) Adsorption and desorption isotherms of the BH, BH-AL2, BH-HT2-AL2, BH-HT6-AL2, and BH-HT24-AL2. (f-j)

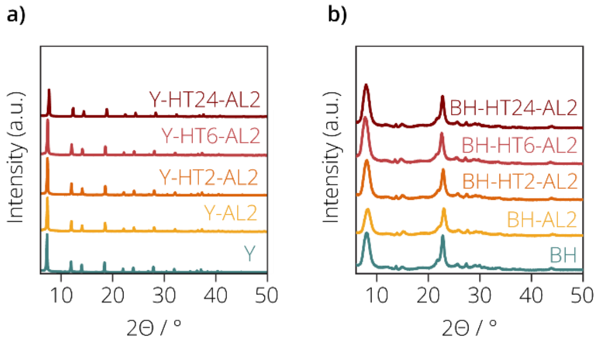

Figure S3. (a) X-ray diffraction (XRD) patterns of the studied zeolite Y materials and (b) studied zeolite Beta materials. In this figure HT signifies hydrothermal treatment and the following number the duration of this treatment in hours. Furthermore, AL2 signifies a 2M nitric acid washing treatment.

Table S2. Results obtained via Rietveld refinement of the Y zeolite diffractograms.

Sample	a_0 (Å)	wR	RF^2	RF	number of reflections
Y	24.32	6.872	2.438	2.453	53
Y-AL2	24.22	8.235	6.728	6.633	50
Y-HT2-AL2	24.23	8.545	7.436	7.387	53
Y-HT6-AL2	24.24	9.231	8.722	6.694	50
Y-HT24-AL2	24.26	8.907	9.510	6.084	50

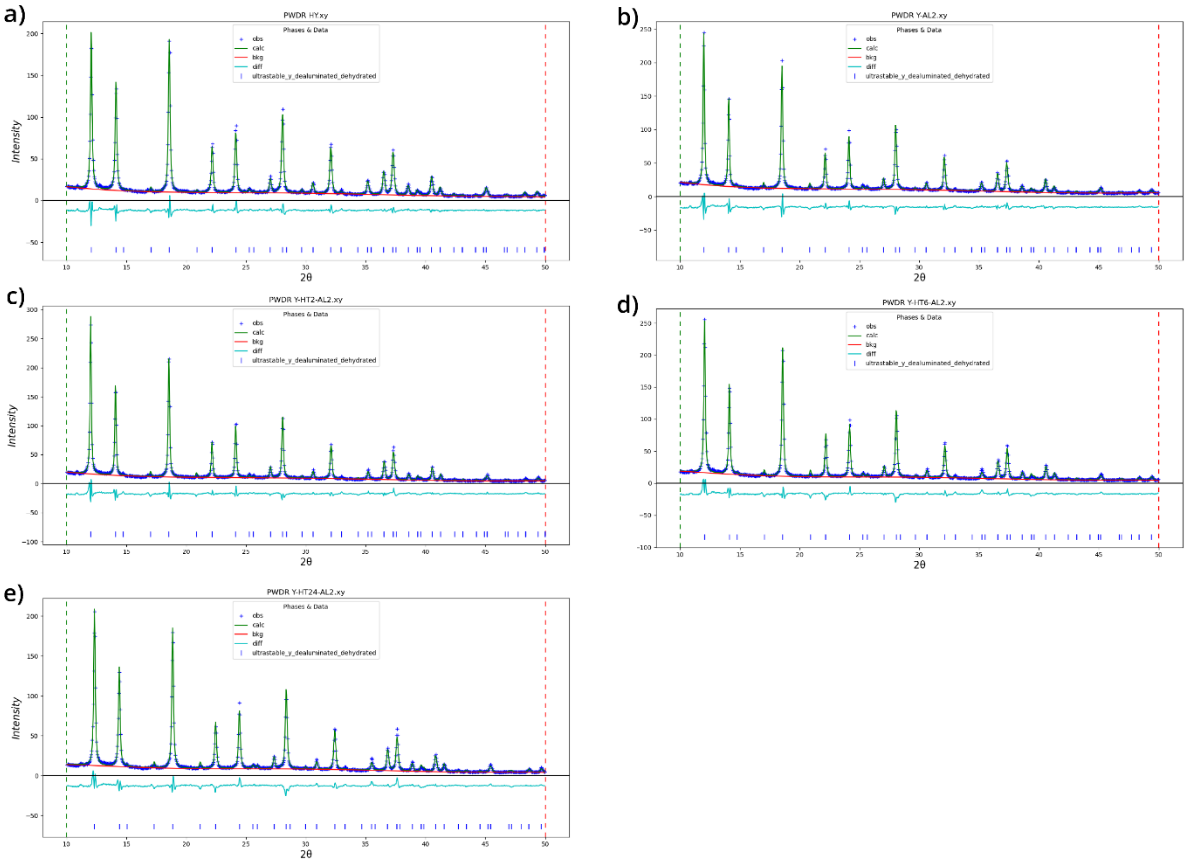

3. RESULTS OF THE ANALYSIS OF REACTANTS AND REACTION PRODUCTS

Table S3. Lactone concentrations (in weight percentage) determined in all the samples taken during the described experiments.

	Lactone concentration (wt.%)									
Time (h)	Y	Y-AL2	Y-HT2-AL2	Y-HT6-AL2	Y-HT24-AL2	BH	BH-AL2	BH-HT2-AL2	BH-HT6-AL2	BH-HT24-AL2
-1 (feed)	2,30	2,42	2,39	2,37	2,29	2,38	2,32	2,32	2,32	2,32
0	1,52	2,22	2,22	2,19	2,20	1,74	1,20	1,07	1,31	1,11
1	0,46	2,02	2,15	2,16	2,16	0,92	0,42	0,23	0,27	0,25
2	0,08	1,92	2,08	2,11	2,08	0,48	0,12	0,00	0,00	0,00
3	0,00	1,80	1,99	2,07	2,02	0,25	0,00	0,00	0,00	0,00
4	0,00	1,70	1,86	2,05	2,01	0,09	0,00	0,00	0,00	0,00
5	0,00	1,62	1,74	-	1,96	0,01	0,00	0,00	0,00	0,00

Table S4. Analyses performed of the reaction products, including acid value, saponification value, iodine value, monomeric fraction and the aluminium content, for the different catalytic tests performed.

Sample name, time	Acid value (mgKOH/g)	Saponification value (mgKOH/g)	Iodine value (g/100g)	Monomeric fraction by HT-GC (%)	Aluminium content (ppm)
Y, t=3h	191.8	192.90	0.03	99.44	6
Y-AL2, t=5h	188.3	192.80	0.07	99.26	0.2
Y-HT2-AL2, t=5h	186.8	193.90	0.05	99.18	0.1
Y-HT6-AL2, t=4h	187.9	196.20	0.06	99.26	0.1
Y-HT24-AL2, t=5h	187.73	193.62	0.05	99.22	0.1
BH, t=5h	191.2	193.80	0.05	99.45	18
BH-AL2, t=3h	192.17	193.28	0.05	99.54	0.3
BH-HT2-AL2, t=3h	191.99	192.7	0.05	99.41	0.1
BH-HT6-AL2, t=3h	192.06	192.97	0.16	99.41	0.1
BH-HT24-AL2, t=3h	191.97	192.93	0.04	99.43	0.1

4. PROCEDURES FOR THE ANALYSIS OF REACTANTS AND REACTION PRODUCTS

The lactone content (wt. %) in the reaction product was determined using an ABB MB3000 IR spectroscope equipped with the PIKE GladiATR (diamond crystal, single reflection) attenuated total reflection (ATR) attachment. The absorbance in the IR spectrum at 1784 cm^{-1} was used to monitor the lactone concentration in the sample at 35 °C. A calibration line of the absorbance and lactone content in the range 0 – 2 % lactone was obtained by spiking 10 different amounts of γ-decalactone (> 98 %, Sigma-Aldrich) in oleic acid (Priolene 6907, Cargill). γ-octadecalactone (made in house at Cargill, 95%) was used to correct the molar absorption coefficient of γ-decalactone.

The iodine value (IV) is a measure of unsaturation and is expressed as the number of grams of iodine consumed by reaction with 100 g of sample under the defined test conditions. To determine the iodine value, about 1.5 g of fatty acid sample was dissolved in a 20 mL 1:1 vol/vol cyclohexane (Merck, ≥99.5 %)/acetic acid (alkalimetric, ≥99.8 %) solution on a hot

plate. After the sample had fully dissolved, 10 mL 3 % magnesium acetate (Thermoscientific, 98 %) in acetic acid was added. The solution was then placed in a Metrohm USB sample processor. The sample was mixed with 10 mL Wijs reagent (iodine monochloride in acetic acid (Merck, 0,1 M) to halogenate the double bonds with magnesium acetate (origin and purity?) as catalyst. The excess of iodine monochloride was reduced to free iodine by adding 10 mL potassium iodide (100 g/L) solution and 50 mL Milli-Q water. The free iodine was then measured by potentiometric titration with sodium thiosulfate (VWR chemicals, 0,1 M).

About 100 mg of the product was weighed in a glass vial for gas chromatography (GC) analysis. Methylation of the fatty acids by BF_3/methanol (VWR chemicals, 99,8 %) was carried out using a Gerstel MPS Dual Head setup. A sample was extracted using heptane (Merck, ≥99.3 %) and sodium chloride (Merck, ≥ 99.5 %) and injected in the following chromatographs: High temperature GC was carried out to determine the concentration of monomeric and oligomeric fractions. The GC was equipped with a metal column with a non-polar stationary phase Cp-SimDist Ultimetal (Chrompack WCOT, 5 m x 0.53 mm x 0.17 µm), a cold on-column injector and a Flame Ionisation Detector (FID). The carrier gas was H_2 with a constant flow of 10 mL/min. The temperature program used was: initial temperature of 60 °C, hold for 1 min; temperature increase of 30 °C/min until 150 °C; hold 0 min; temperature increase of 12 °C/min until 375 °C; hold 10 min. The detector was set at 375 °C. The injection volume was 1 µL for a concentration of 8 mg/mL. The monomer fractions were analysed by GC equipped with a fused silica capillary column with a polar stationary phase FFAP-CB (Chrompack WCOT, 25 m x 0.32 mm x 0.30 µm), a split injection (constant pressure of 15.6 psi and split ratio of 1:15) and an FID. The carrier gas was H_2 with a constant pressure of ~15.3 psi and a split flow of ~153 mL/min. The temperature program used was: initial temperature of 120 °C, hold for 0 min; temperature increase of 8 °C/min until 250 °C; hold 20 min. The detector was set at 250 °C. The injection volume was 1 µl for a concentration of 8 mg/mL.

The acid value was determined by dissolving 1.6 g of sample in 25 mL of ethanol (96 %). The solution was titrated with a KOH (VWR chemicals) solution (0.5 M) until neutralised and the acid value was then determined as the amount of mg KOH reacted per g of sample.

In addition to the acid value, a saponification value was determined by boiling 1.6 g of sample in an excess of KOH solution (20 mL, 0.5 M) and subsequent colorimetric titration with HCl (VWR chemicals, 0.499-0.501 M) solution until it was neutralized, using phenolphthalein as indicator. The saponification value is derived from the volume used for the titration after subtracting the volume found for the blank. The extent of aluminium leaching from the zeolite into the product was determined with Inductively Coupled Plasma-Mass Spectrometry (ICP-MS) on an Agilent 7800 mass spectrometer, equipped with a Pt ShieldTorch system and an Agilent micromist nebulizer. Firstly, 0.5 g of sample was dissolved in 10 mL 70 % HNO_3 (J.T. Baker, 69.0-70.0 %) and placed in a closed vessel microwave (CEM SPD-80) for digestion (max temperature of 200 °C). After completion, the sample was diluted to 50 mL with Milli-Q water. Analysis was based on external calibration, by spiking the blank solution with at least 6 levels of an aluminium internal standard (AccuStandard, 1000 µg/mL in 2-5 % nitric acid). Helium was used as collision gas, aluminium was measured at m/z 27 and an internal standard of germanium (AccuStandard, 1000 µg/mL in water, tr hydrofluoric acid) was measured at m/z 72.
